# Taking after a parent: Phenotypic resemblance and the professional familialisation of genomics

**DOI:** 10.1111/1467-9566.13699

**Published:** 2023-08-15

**Authors:** Adam Hedgecoe, Kathleen Job, Angus Clarke

**Affiliations:** 1 School of Social Sciences, Cardiff University, Cardiff, UK; 2 Institute of Medical Genetics, Cardiff University, Cardiff, UK

**Keywords:** clinical decision making, ethnography, genomics, uncertainty

## Abstract

This article draws on 2 years’ worth of ethnographic observation of team meetings to explore decision-making in an NHS clinical genomics service. The focus of discussions was on ambiguous genomic results known as VUS or Variants of Uncertain Significance, which may be pathogenic but which also may turn out to be benign. In examining decision-making around such results, we note how, in contrast to much policy and promotional material in this area, clinicians in these meetings (clinical geneticists and genetic counsellors) place great emphasis on parental phenotypes and whether the parents of a patient share the symptoms and signs of the suspected condition. This information is then combined with the result of genomic tests to decide whether the variant a patient has is responsible for their condition. This article explores the way in which clinicians attempt to flexibly enrol parents into genomic explanations through informal diagnosis of their possible phenotypes and the way in which actually meeting parents allows some clinicians to trump explanations based on documentary or photographic data. The paper sheds light on the way that earlier scholarly understandings of such decisions (around, say dysmorphology) remain relevant and explores claims that laboratory tests overrule clinical decision-making.

## Introduction

The past 5 years have seen an acceleration in the movement of new genomic technologies—what might be generically referred to as Next Generation Sequencing (NGS)—into NHS clinical practice, with activities such as the ‘100,000 Genomes Project’, the development of a NHS Genomic Medicine Service and high-profile publications such as the 2016 Chief Medical Officer’s Annual report, *Generation Genome* (Davies, 2017; Prime Minister’s Office, 2012; Robinson, 2020) underlining the financial investment and structural changes taking place.

The umbrella term, ‘Next Generation Sequencing’, covers a number of different ways of looking at a person’s genome—their complete set of genetic information contained in their chromosomes—which tend to share parallel investigation of large amounts of genomic materials, and which can be best distinguished in terms of the scale at which they work. The narrowest approach is offered by ‘gene panels’ which involve the sequencing of a pre-determined set of genes, usually grouped together around specific conditions (for example, a ‘cardiomyopathy panel’). Broadening outwards is whole exome sequencing (WES), which focuses on the 1%–2% of the genome that codes for protein (the exome) and which is estimated to contain around 85% of disease-causing mutations. This is seen as a cheaper—but also more clinically relevant—alternative to whole-genome sequencing (WGS), where a person’s whole genome is sequenced. Finally, in addition to these approaches, we might add more structural assessments of the genome, such as array comparative genomic hybridisation which, while not employing sequencing, provides a genome-wide review of the number of copies of chromosomal elements.

As we might expect, the introduction of such technologies into clinical practice raises several challenges and has proved a site of intense interest for medical sociologists and anthropologists, STS scholars and other interested outsiders to the clinic. While clinical genetics has been a long-standing area of interest for medical sociology (Richards, 1993), especially with regard to the way in which clinical decisions around genetics are presented to patients/parents, we know far less about how modern genomic technologies, of the kind which are promoted by the NHS Genomic Medicine Service, are incorporated into clinical practice. On the one hand is a perspective rooted in previous scholarship—such as the use of pedigree diagrams to explore the nature of ‘family’ and inheritance (e.g. Atkinson et al., 2001) or clinicians’ flexible, contingent attitude towards the application of clinical testing guidelines (Wood et al., 2003)—which emphasises the limited impact of genetic testing on clinical practice:

Even though one might expect genetic testing to trump other forms of clinical knowledge, this does not seem to occur: not even for those conditions that are seen as inherently genetic. The clinical judgement still remains central and the authority of doctors lies in the art of immediately “seeing” a look that matches a diagnosis and subsequently directing further testing (genetic or non-genetic).

(Kuiper et al., 2021, pp. 437–438)

In contrast to this is a position that suggests that, as they move into practice, new genetic technologies will displace clinical judgement from the genomic clinic. Such a position is gaining ground in STS and the social studies of genomics and moves from ‘studies that show how [some] new diagnostic categories and diseases are produced not by clinical judgement but by molecular technologies’ (Skinner et al., 2016, p. 1314) to the position that:

non-clinical, laboratory-based results increasingly tend to dictate, rather than simply contribute to clinical decisions, and by the same token encroach upon what was once the uncontested domain of the clinician, namely clinical decision-making.

(Bourret et al., 2011, p. 816. See also Rabeharisoa & Bourret, 2009)

It would be a mistake both to claim that such arguments are dominant in the literature and that this work focuses solely on clinical practice, covering as it does a range of activities from the ‘genomic designation’ of new medical syndromes (Navon, 2011), to the reshaping of regulations via the development of pharmacogenomics (Hogarth, 2012) and the interaction between health infrastructures and genomic technologies (Aarden, 2016). However, there remains a specific thread arguing that ‘by redefining clinical-laboratory interfaces, genomics is simultaneously redefining key features of medical work’ noting that ‘a new wave of STS investigations have provided clear evidence of change in clinical practice driven by the adoption of genomic technologies’ (Cambrosio et al., 2018, p. 144). While acknowledging that there is a complex inter-relation between genomic technologies and clinical practice, the re-shaping of clinical decision-making along genomic lines lie at the heart of this approach.

To investigate this tension, we explore the impact of genomic testing on clinical practice by looking at how groups of professionals make decisions about uncertain genomic results—the kind of result known as a VUS, a ‘Variant of Uncertain (or sometimes Unknown) Significance’, those sections of DNA, the value of which—benign or pathogenic—is unknown at the time of testing (Frebourg, 2014). While these variants have been acknowledged for around 20 years (Federici & Soddu, 2020), the significant increase in genomic information produced by Next Generation Sequencing technologies means that, in each case tested, the number of VUS has also expanded, producing considerable challenges for clinical staff who are tasked with interpreting this data (Domchek & Weber, 2008; Kim et al., 2019). The key point to consider is that while clinical genetics has a long history of professional decision-making and communication of results around *probabilistic* uncertainties—the uncertainty of the BRCA1 positive woman, at increased risk of disease, not knowing whether she is going to develop breast cancer, for example—VUSs present more epistemic challenges, of whether the variant identified is related to a specific condition and thus whether there is an increased risk at all (Timmermans et al., 2017).

Previous scholarship has given us some insight into how VUS—which while unknown, are deemed potentially suspicious—are dealt with throughout the diagnostic pathway (Kuiper et al., 2022) with a particular focus on how even those tests with little or no direct impact on clinical interventions are made ‘actionable’ for patients and their parents (Stivers & Timmermans, 2017); how professionals use VUS results as resources for the clinical collective (Timmermans et al., 2017); and how, in clinical consultations, genomic uncertainty is resolved out of the interaction between clinicians and patients’ parents (Stivers and Timmermans, 2016). Building on previous work on the role that various standards (Timmermans, 2015) or the continuing importance of single-gene approaches to disease (Timmermans, 2017) play in the shift of clinical exome sequencing (CES) into the clinic, this paper explores how professionals make decisions about VUS and other sequencing results prior to discussion with patients or their parents. This article sheds light on some aspects of this decision-making, showing how genetics professionals engage in ‘the familialisation of genomics’ by drawing on—or over-interpreting—family characteristics in their analysis of genomic data.

In keeping with previous scholarship exploring the nature of clinical decision-making in the genetics clinic, our data underlines the importance of both visual observation of phenotypes—that ‘the routine ritual inspection of photographic images of patients holds a central place in diagnostic work’ (Shaw, 2003, p. 40)—and clinical decision-making beyond the individual genetic test result. In addition, we did not find that decision-making about VUS varied depending on the kind of testing technologies used in the various settings observed. In part, this is because in the NHS, most WGS and WES take the form of ‘virtual panels’, where interpretation is focused on a number of areas associated with the phenotype in question. While the whole exome or genome sequence will have been generated, only the specific, pre-selected sequence elements are interpreted. The technological source of these elements (which may be classed as VUS) loses relevance.

This article focuses discussion of decision-making around phenotypes (visual and otherwise) away from the affected patient and onto those of the parents. In modern NHS, genetics clinics—even those making use of advanced testing technologies, such as Whole Exome or WGS, it is quite standard practice to include family information, including parental phenotypes and genotypes. For example, the exome sequencing that the centre had access to (via a research project) tended to test trios (i.e., the proband child plus parents) rather than singleton probands on their own, and trios are preferred in the rare disease context. However, if one or both parents are not available, then testing will go ahead with WES/WGS anyway, accepting that interpreting VUSs becomes much more difficult. In contrast, in the context of cancer, when you are often dealing with adult patients, it becomes much more difficult to get samples from the adult’s parents, especially if one of them has died of cancer.

The role of parental sequencing data is twofold, depending on whether one ignores or identifies variants inherited from parents. A common initial approach to exome analysis would be to focus on de novo variants (i.e., present in neither parent) and recessive variants (i.e., genes with both alleles containing variants, one inherited from each parent) but to ignore inherited heterozygous variants (when only one copy of the gene has a variant and that is also found in a parent).

The alternative approach, and a focus for our work here, which involves including inherited variants, involves much more work for the lab and the clinical teams, who need to look at the evidence for and against pathogenicity for each of the large number of such variants found in everyone. Thus whether a resemblance between parent and child is simply that—a ‘normal’ familial resemblance—or is related in some way to a shared pathogenic variant is a crucial decision for both the lab staff and the clinical team, especially given that the effects of variants in some genes can be very variable, highlighting the challenge of working this out.

The remaining parts of this study explore aspects of the role the parental phenotype plays on decision-making around VUS by examining the strong cultural role that the parental phenotype plays in deciding on testing; the complex and flexible way in which parental phenotypes are interpreted, which in turn leads onto the speculative way in which such phenotypes are sometimes ‘invented’ to aid the resolution of a decision; and the way in which the participants’ views about such phenotypes do not carry equal weight.

## Methods

This article presents data from a Wellcome Trust funded project, ‘Professional decision-making around next generation clinical genetics’, which set out to explore the way in which groups of professionals make decisions about uncertain aspects of modern clinical genetics and how those uncertainties are communicated to patients.

Our approach was ethnographic, sitting in on and audio recording professional meetings across a number of different settings over roughly a 2-year period (September 2017 to November 2019) (see Table 1). Meetings took place in the same single institution—anonymised as ‘Ernshire’—in various settings and varied in terms of frequency (from weekly prenatal to the bi- or tri-monthly neuropsychiatric genetics meetings), usually lasting between one and 2 h.

The choice of meetings to observe was driven partly by our focus on genomic testing technologies (e.g., Genomics multidisciplinary team [MDT]) and partly by an interest in a range of different conditions (i.e., cancer and inherited heart disease).^1^ The nature of the conditions being tested and the organisation of the service mean that the same case might be presented at a number of these meetings. For example, parents might enter discussion via the prenatal meetings with their genetic results being subsequently discussed at both dysmorphology and Genomics MDTs.

The majority of participants in these meetings—mainly Consultant and registrar clinical geneticists and genetic counsellors but also some staff from the diagnostic laboratory—came from the large local NHS genetics service and, while attendance varied, membership of the different meetings overlapped considerably, with the same people attending a number of them in any 1 week.

Analysis of our data was an extended, iterative process, starting with cases of interest being flagged as such by KJ (who observed the majority of the meetings) at the point of recording. They were then transcribed and analysed by AH before further discussion took place within the team. AC contributed to this analysis and co-wrote the paper with AH and, while our broad analytic focus was on VUS and the discussions surrounding them, specific areas of interest, such as the focus of this article, arose out of our data and were not identified in advance of the analysis.

## Results

### The importance of parental phenotype

In this context, a clinician’s visual assessment is not just of the *patient’s* phenotype—the signs and symptoms they have that indicate a specific genetic cause—but also that of one or both of their parents. Looking for shared characteristics is a sensible thing to do if you are talking about potential genetic causation: lack of overlap may indicate a de novo variation in the child and slightly different symptoms in a parent may nudge the diagnosis in a different direction or lead to an overall reassessment.

Take, for example, a case presented by a trainee clinical geneticist, James, concerning a 2-year-old boy with an unusually large head and some developmental delay (especially around speech), whose mother also has a large head, though no developmental delay or other symptoms. Following testing using a gene panel (i.e., sequencing of a specific set of predetermined genes associated, in this case, with overgrowth and intellectual disability), the result was a VUS in the *PTEN* gene, where pathogenic variants are normally associated with Cowden syndrome, a condition linked to benign tumours called hamartomas and increased risk of various cancers, including breast and uterine, although developmental delay and increased head size are possible aspects of the phenotype. As a VUS, this variant is classed as a ‘3’, sitting in the middle of a five-point scale ranging from Benign (1) and Likely Benign (2) through VUS to Likely Pathogenic (4) and Pathogenic (5). The role of the discussion is to marshal and assess the various available forms of evidence (of differing strength) and combine them to move the classification up into the realms of pathogenic or down to make it benign and thus not relevant for further discussion (For a detailed analysis of this process see Hedgecoe et al., 2023).

One option to resolve the uncertainty around this VUS and allow it to be reported as pathological is simply to test the mother, to see whether she carries the same variant. James’s suspicion is that this variant is de novo (i.e., a new mutation in the boy, not inherited from either parent), and if the mother does not carry this variant then this will be confirmed. The formal criteria used to help decide whether a variant is pathogenic or not, draughted by the American College of Medical Genetics and Genomics and the Association for Molecular Pathology (Richards et al., 2015), assign some explanatory value to de novo status, and thus in this case, could be combined with other aspects of this case to allow James to classify this VUS as pathogenic. From this perspective, the actual parental phenotype is a bit of a red herring, with James suggesting that *‘if we’re trying to fit it* [that is, the variant] *to a phenotype, that’s not really the question in hand’*. The point is that in terms of the formal assessment criteria, in this case, parental phenotype will not help much:

So whether you find out whether it, you know, if it’s come from Dad, it’s still going to be a VUS, but we’re just going to be looking at dad’s side of the family as opposed to mum albeit that, I don’t know what his head size is, but it wouldn’t explain mum’s head size. And indeed the head size might just be a trait that’s come down mum’s side of the family, but it wouldn’t categorize it as being anything other than a VUS.

(James)

But other clinicians push an alternative approach, emphasising the importance of the mother’s phenotype in an accurate assessment of the variant. This is clear later on in the discussion where Alison, a genetic counsellor, suggests that finding out that the mother carries the variant would allow a different explanatory criterion to be applied: *‘If mum’s got it, could you say ‘segregating with phenotype’?’*. James’ sceptical response—*’What phenotype?’*—prompts Mary, one of the more experienced clinicians in the room, to map out this alternative approach:

we currently think she doesn’t have a phenotype, but as Alison says, we don’t actually know that, so we haven’t specifically gone through the diagnostic criteria for Cowden in her and checked them. So I think I think we do need to do that and make sure that she doesn’t have a phenotype. You know, we haven’t looked in her mouth.^2^

James: No

Mary: You know, so she may suddenly have a really good phenotype, and we’ll all go ‘oh look she’s got…’

While discussion continues, it consistently circles back to the need to confirm the mother’s phenotype, with James’s explanation of the situation regarding the value of a de novo result in terms of the formal criteria—’*We only need a supporting* [criterion], *we’ve got two moderates and a supporting’*—being met with an account of the limits of such an explanation from Bill, a consultant clinical geneticist: ‘*if it were* de novo, *but then that wouldn’t explain mum’s head shape’* followed by Bob, another clinical geneticist, offering his opinion:

I think after phenotyping parents, if they don’t have any definite phenotype, I think that we would offer testing with an explanation that see if the only way it will be helpful if is if it is *de novo* in the child. If one of the parents carry it [i.e. the variant], we probably will still be in the VUS situation. We have to explain this.

Alison: Unless unless one of them has got…

Bob: yeah, unless one of them has got [phenotypic] features.

In this resolution of what to do next, the team makes clear that testing the parents only makes sense in the context of their phenotype. While the easiest approach is to just test the mother and, if she does not carry the variant, assume it is a de novo variant in the patient, this does not sit well with the meeting. Such testing should only happen once a proper phenotypic assessment of the parents has taken place and the familial context has been clarified. In this context, the parental phenotype serves a gatekeeping role with regard to testing the parent.

In terms of the impact of genetic testing on clinical decision-making, this case highlights how developments in genomic testing—in terms of accuracy, for example—have not necessarily led to laboratory tests taking precedence over clinical decision-making. In the past, a key distinction could be made between ‘making it familial’ (tracing specific physical features across different generations using photographs and family trees) and ‘making it genetic’ (linking these features to specific, identified molecular change). As set out by Latimer and colleagues, the challenges and uncertainties around ‘making it genetic’ (and hence the need for deferral of a decision and the retention of uncertain cases within the genetics service) depend in large part on the nature of the tests being run and their (in)adequacy to confirm a genetic diagnosis (Latimer et al., 2006).

In our data, with the subsequent development of testing technologies, the lack of clarity about the existence (or not) of a variant in the *PTEN* gene has largely gone, yet the need to ‘make it familial’ remains, in order to decide whether the patient has a genetic condition. In large part, this is because of the way in which the rules around deciding whether a VUS can be re-classified ‘add up’ various criteria (for example, whether a phenotype can be said to be present in other family members) to reach a decision (see tab. 5, Richards et al., 2015, p. 414). In this case, since the familial status (or not) of the phenotype is the last link in the decision-making chain, it serves as the deciding factor. New uncertainties have arisen out of the sequencing panel, leading to a new uncertainty, the VUS.

### The flexibility of parental phenotype

As we might expect, in clinical genomics, some of the strongest insight into the importance of parental phenotypes in professional decision-making comes not from those cases where such information is smoothly negotiated but rather in those examples where agreement over the value of this information is harder to reach.

The complexities of these kinds of negotiations can be seen in a case involving a 14-month-old girl who, for various reasons, is sequenced using a gene panel which indicates she carries a mutation in *MECP2*, the gene associated with Rett syndrome, a rare developmental disorder of the brain. Initially, the diagnosing clinical geneticist resists the Rett’s diagnosis provided by the paediatrician, since the child does not display Rett-specific symptoms (such as the well-documented ‘regression’ phase), and *MECP2* is not associated with other symptoms the patient has.^3^

Over the course of the initial discussion, the team agrees that, despite this poor fit with the child’s phenotype, Rett syndrome is the best diagnosis, with Alan, the lead clinician on this case, suggesting that the patient looks ‘*mildly Rett-y*’. The variant in question is classed as a VUS although it is ‘*in a known [mutation] hotspot so it’s I think it’s not—this particular one has not been reported before—but I think it’s very similar to those other ones that were reported as pathogenic*’. Although de novo mutations in *MECP2* are not uncommon, the obvious way to help clarify whether or not the variant the child is carrying is causing her symptoms is to test the parents, to see whether it has been inherited from either of them. Which is where further problems begin to arise.

Upon testing, it becomes clear that the child’s mother also carries this variant of the *MECP2* gene and should, thus, (if it is pathogenic) have the same kind of difficulties as her daughter. Since some variants in *MECP2* can lead to a degree of simple cognitive impairment in males who carry these variants but without the features of Rett syndrome, this is likely to occur also in some females who carry such variants, although they would usually be affected less (more subtly) than the males. Thus, the question is, can the mother be thought to have a cognitive impairment?

Bill: So… but she’s *[i.e. the daughter]* behaving in a Rett-like manner?

Alan: Yeah so she is behaving in a Rett-like… but mum who has the same mutation is educationally normal – she works in Frasier’s *[a well-known chain coffee shop]*, she’s getting on with life, she’s planning a wedding soon.

Michael: If you find it in her [the mother’s] dad or another male in the [mother’s side of the] family then obviously that’s really helpful.

If this variant is the cause of the child’s phenotype, the challenge now is how to explain the child’s symptoms and the mother’s apparent *lack* of problems. This exchange between three clinical geneticists sets out one possible solution—further familial testing, which might identify other carriers in the family, which (especially if they are male) would weigh against the variant being of any relevance. But conversation swiftly turns to a possible technical explanation for the discrepancy between the mother and child’s phenotypes, the possibility of skewing of the process of X chromosome inactivation (XCI). XCI is a biological process in females in which one of the two X chromosomes in each cell in a female is inactivated, and the choice as to which X is inactivated is usually random, with approximately half of the cells inactivating one X chromosome and the other cells inactivating the other. However, in some females, the same X is inactivated in all or nearly all cells, and this can mask the effects of an alteration in a gene on that copy of the X chromosome because only the other (normal) copy of the gene is expressed. Although this would be most unusual, it means that an unaffected mother could carry a *MECP2* variant on her inactivated X chromosome without it manifesting, only to pass it down to a daughter who could then be affected, if she has the usual pattern of random XCI (BIOS Consortium et al., 2019; Knudsen et al., 2006). As the details of this possible solution are mapped out, discussion returns to the possibility that the patient’s mother might have a Rett syndrome phenotype:

Bill: Yes I’m just wondering whether you could have a situation where mum has it in her white blood cells but actually that’s because the mosaic cloning appears to have taken over as it were and that actually if we tested other tissue in mum…^4^

Alan: Wait a sec, I think the only way we’re getting into that is if I test the grandparents and they don’t have it we could say well maybe one of the protective mechanisms in mum is that you know she’s not fully constitutionally heterozygote.

Bill: Yeah

Andrea: and mum doesn’t have any other problems?

Alan: she’s obviously she hasn’t gone to university so she’s got kind of low average intelligence but she’s not Rett-y – no seizures

Bob: Are they coping with these children?^5^

Alan: Yes

While there is clearly a tonal difference between Alan’s two descriptions of the mother as:

educationally normal – she works in Frasier’s, she’s getting on with life, she’s planning a wedding soon

and

she’s obviously she hasn’t gone to university so she’s got kind of low average intelligence.

they are both compatible with, ‘*she’s not Rett-y – no seizures’*. The evidence is clearly not strong enough to claim that the mother has even a mild version of Rett’s syndrome. Add to this the fact that the mother is ‘coping’, and the case for a mild form of Rett’s syndrome in the mother (and thus in the daughter and thus a causative role for the variant) has not, yet, been made. What is being unsuccessfully attempted here is the diagnostic technique known as ‘expanding the phenotype’—where ‘genes are given primacy in what the phenotype should be rather than what is reported in the patient or known about a condition’ (Timmermans, 2017, p. 163)—as not just applied to patients themselves but to members of their families. For example, Timmermans discusses a case of a child with a good fit between a variant for a dominant condition (optic atrophy) and their phenotype, where the clinical team provisionally diagnose the child’s father (who also carries the variant) with a very mild, late onset form of the same eye condition. This, despite the lack of clinical evidence that the father had problems with his eyes ‘In essence, the team decides that if the father has optic atrophy, then the variant explains the son’s diagnosis. The team thus suspected a disease in a person who was not even their patient’ (Timmermans, 2017, p. 163).

In the Rett syndrome case, while the possibility of expanding the putative phenotype associated with this variant—to include the ‘kind of low average intelligence’ of the mother—is discussed, this particular approach is not adopted at this point. Instead, the team decides to move forward with testing for skewing of XCI and trying to find out more about the males in the mother’s family, solutions which would lessen the need to decide whether the mother has a form of Rett’s.

Five months later, the case returns to the Dysmorphology meeting and Alan brings the team up to date:

so the issue was that mum has the same MECP2 frameshift mutation – it’s not in her *[the patient’s mother’s]* mother, so it’s not in the maternal grandmother and the family are reticent to contact mum’s dad – the grandfather – because he’s, yeah, they don’t really have much social contact with him. We’ve done X-inactivation in the mum; mum is not skewed, she’s just, there’s no obvious skewing there.

Thus the two solutions set out in the previous meeting—tracking the gene back through male members of the maternal family and testing for X-inactivation—are stymied by both biological and social impediments: The gene does not appear to be skewed in the patient’s mother, and the family have no contact with the patient’s grandfather.

In this meeting and in subsequent discussions at a Genomics MDT meeting 8 months later, colleagues coming to this case for the first time clearly attempt to ‘expand the phenotype’ in order to count the mother’s ‘low end of normal’ intelligence as indicating a mild form of Rett’s syndrome.

**Table:** 

Dysmorphology meeting	Genomics MDT meeting
*James: and mum doesn’t have a phen*[otype]… *mum has no education*… *Alan: Mum works, mum works in Frasier’s, went to a normal school* *James: So mild*… *Alan: So she’s kind of low end of normal* *Bob: she didn’t need any extra help or anything in school*	*Jane: How is mum? Generally?* *Alan: so mum has sort of low normal intelligence: she did GCSEs and went to work in a coffee shop, so she holds a steady job.* *Jane: so mild*… *sort of* *Alan: well she could be*… *so she’s done GCSEs and been in a mainstream school so I wouldn’t say she’s intellectually disabled - I’d say she’s within the normal range of intelligence but kind of low normal intelligence*… *so*

In each case, a colleague—James or Jane—who had not been at the earlier meetings where this case was discussed, tries to work through the evidence. In each case, upon being told that the mother ‘*works in Frasier’s, went to a normal school’* or ‘*did GCSEs and went to work in a coffee shop, so she holds a steady job’*, the colleague in question offers an account, albeit tentative, of these features in terms of a mild form of intellectual disability (and hence, perhaps, an unusually mild version of Rett syndrome). In each case, Alan and/or Bob, professionals more familiar with the details of this case, correct this misinterpretation: ‘*she’s kind of low end of normal…she didn’t need any extra help or anything in school’* or *‘she’s done GCSEs and been in a mainstream school so I wouldn’t say she’s intellectually disabled’*.

Because the logic at play here, with all other things being equal, requires a phenotypic similarity between a parent and a child who both carry the same variant, professionals’ initial response to a case is to ‘familialise’ it, to look for a connection between the phenotypes of family members, in this case, by expanding the phenotype. In the end, in this example, the familialisation of genomics through phenotypic resemblance, which is such a fundamental aspect of how professionals begin to make decisions about VUS, is rejected by the team. But, the intuitive appeal of this approach is clear.

### Inventing parental phenotypes

The appeal of parental phenotypes is such that even in cases where no information is available regarding biological parents, speculative or fictitious parental data is drawn on. A good example comes from the case of a 6-year old girl who presents to the service with the kind of mixture of symptoms commonly seen by these experts:

kind of global developmental delay…She’s got a mild hypertonia,^6^ some vacant spells but I don’t think she’s had an EEG. Fine motor is a bit off, speech delay, very sociable, slightly immature behaviour. She goes to a mainstream school but she’s got a statement *[of special education needs]*. There is this question of whether she’s having absence seizures, she’s a little bit dysmorphic with her epicanthic folds….

(Alan)

She is tested via a pilot study that is investigating the use of CES, but, unusually for such cases, she is not tested along with her parents (a ‘trio’) but rather on her own. After a moment’s confusion, Alan interjects:

a key piece of information that I just remembered is that she’s adopted. Which is why she is a singleton *[i.e. not tested with her parents]*. I was confused because her adopted parents have the same surname, but I think that’s changed since she’s been adopted. So obviously one or both of these mutations could be inherited from affected parents who also have learning problems and have not coped with life because of that mutation.

Alan’s speculation about affected biological parents with learning problems, with those (genetically derived) problems being the source of the adoption (they ‘have not coped with life because of that mutation’) fits within a broader experience for these professionals that adoption is often related to ‘not coping’ that, in turn, is often associated with drugs or alcohol or psychiatric disease or cognitive limitation. Thus, while not open to confirmation, this example of familialisation is, from this perspective, entirely reasonable.

Another case of speculative parental phenotyping comes in the discussion around a woman who collapsed with a dissecting aortic aneurysm—a swelling of the aorta resulting in a tear—and who, upon testing carries two possibly relevant genetic variants. Following a discussion of the pros and cons of each variant, Kerry, who brought the case to the meeting, remains frustrated: ‘*I still don’t know what to bloody do with it! She’s not…she’s got parents - she’s got dad I can test*’. When asked about the patient’s parents, she notes that ‘*Dad is 71 with rheumatoid* [arthritis] *and* [otherwise] *well apparently, mum has already passed away at the age of 46*’, at which point Alan suggests ‘*So mum’s history is suspicious*’. Kerry is not so sure—’*No, well, hhhmm*’—so, Alan starts to construct a putative medical history for the patient’s mother: ‘*So her mother died of renal failure and endocarditis. Now one of the reasons you get—So it could be the endocarditis…But maybe she’s got a dodgy vasculature, which got infected and/or aneurysm*.’ At this point, Kerry objects slightly: ‘*but she had a history of scarlet fever as well*’ to which Alan responds: ‘*true, but maybe she aneurysmed off her renal arteries*’.

These examples underline the importance of parental phenotypes as an explanatory resource in the resolution of genomic VUS. Linking to such phenotypic information is so useful that even these kinds of speculative accounts—which are acknowledged as such—can be enroled into the team’s discussions. They can trigger searches in the medical records of absent or deceased relatives and may contribute to the final decision on a case.

### Policing the parental phenotype—Being there

However useful the familialisation of genomics through phenotypic resemblance is, what becomes clear from our data is that it is not a resource that all members of the meeting can take advantage of. While considerable parental phenotypic information is included in the paper reports, photographs and electronic files that are available to these MDTs, as we might expect from previous scholarship (for example, see Latimer, 2013, p. 91), the experience of actually meeting the parents—and observing their phenotypes at first hand—tends to trump other accounts, to the extent that team members use the authority of first-hand experience of phenotypes to ‘police’ attempts at explanation.

As previous work on the medical profession might suggest (e.g. Halpern, 1992), such policing is based, in part, on medical speciality, especially that of clinical genetics. For example, in a hastily convened meeting to discuss a VUS presented at one of the weekly prenatal meetings, discussion takes a tangential turn when Sally (from the laboratory) and Nicky (a senior consultant clinical geneticist) recall a recent, similar case, involving a young woman and a foetus with an 11q deletion associated with learning disabilities. Sally suggests that *‘I was half expecting her to be maternally inherited, that one’*, that is, that the mother would display some of the characteristics associated with the variant, going on to relate an exchange in the laboratory where (based on the written reports of the mother’ phenotype) one of the other staff refused to accept that the mother was not a carrier: *‘I happened to be in fetal med right, when [name 1] ran in to ask [name 2] what the [unclear] PCR was. And [name 2] is going, ‘yeah it’s here, it’s normal’. [Name 1] on the other hand was going ‘lab has got it wrong, you can’t tell me it’s normal’…She wouldn’t have it’*.

Having already described this mother as *‘a very vulnerable, ill-educated highly strung anxious, girl’*, Nicky goes on to make clear that this does not mean she is displaying the phenotype in question, arguing that the lab colleague [name 1] *‘hasn’t spent time having a proper conversation with this girl. She’s vulnerable, she’s not stupid though. I expected it to be not inherited by either of them. Although she comes across as inadequate, if you spend time with her, she’s just incredibly vulnerable’*. Nicky, as a senior clinician who meets patients and parents, claims authority over these kinds of decisions about parental phenotypes.

While in this case, we might suspect Nicky is involved in some form of policing of professional boundaries between the clinic and the laboratory, it is clear from other examples that the key aspect of this discussion lies in the direct experience of familial phenotypes within clinical genetics. A good case of this can be seen in the discussion around a 17-year-old patient with a range of symptoms who (with her parents) has been exome sequenced and carries three potential causative variants—two paternally inherited and one maternally. One of the paternally inherited variants is quickly dismissed—*’So GRIN2A is associated with epilepsy…and she doesn’t have epilepsy, so we thought that was quite unlikely’*—leaving one variant from her father (in the *KM2TD* gene, mutations of which are associated with Kabuki syndrome) and one from her mother (in the *CREBBP* gene, associated with Rubenstein Taybi syndrome).

For phenotypic reasons—Kabuki syndrome is associated with very specific facial features—discussion centres on the *CREBBP* variant and Rubenstein Taybi, a condition characterised by short stature, moderate to severe intellectual disability, distinctive facial features and broad thumbs and great toes:

Alan: *[reading from the report]* she has broad terminal digits. So, she has several features of Rubenstein Taybi Syndrome. Broad thumbs, relatively broad toes, a long columella^7^ and obviously, cardiac defects which are more common in Rubenstein Taybi as well. I haven’t checked but would imagine deafness as well. *[loads up picture]* Obviously this is mum here.

Simon: she hasn’t got the nose.

Alan: well she’s got a little bit, quite a prominent, bulbous tip, I mean, I guess…

It is at this point that Simon, who met the family when they first attended, challenges the phenotypic link between mother and daughter:

Simon: Certainly when I when I was in the room with them I didn’t look at them as a family and think ‘you have inherited something from one or the other’. I didn’t think there was a particularly striking resemblance.

Undeterred, Alan returns to the photograph of the mother looking for other phenotypic clues: ‘*you see the dental feature of Rubenstein Taybi Syndrome, is, let me get the…Talon cusps*,^8^ *so you could do a little bit of phenotyping on her teeth. You know, is that a talon cusp there?*’ But there is not enough visual information in the picture to make the phenotypic link on the basis of the mother’s teeth, and Simon, drawing on the authority generated from having met the family in person, has closed off other possible phenotypic links. Given that both Alan and Simon are clinical geneticists, Simon’s authority over this explanation lies not in a hierarchy of specialisms (e.g., the clinic vs. the lab) but in the authority generated from actually meeting the family and from being able to observe familial similarities and potential phenotypes in the flesh.

## Discussion

The key insight of this paper—that in the modern genomic clinic, parental phenotypes (and genotypes) play a crucial role in decision-making—is a point of interest given the relentlessly individualistic language and tone of most UK policy debate in this area. These discussions focus on the risk that specific variants might pose to individual patients, with a rhetoric of screening individuals and the movement towards some kind of personalised health care. Even those discussions where families are mentioned—for example, the UK Government report *GENOME UK: The future of health care*—tend to focus on the value of an individual’s genomic test to other members of their family (UK Gov., 2020). What is missing from these discussions is the very real need to clinically engage with other members of a family (normally parents) to work out whether the genomic variant in question is pathological or not in the first place.

Thinking more broadly about the familialisation of genomics, it is interesting to look at parallel literature exploring the ways in which parents respond to their children being diagnosed with a genetic condition. There are strong echoes of professionals’ familialisation of genomics in *lay people’s* explanatory mobilisation of family information in the genetics clinic. Discussions of family resemblance—who looks like whom—and the inheritance of visible characteristics is a run of the mill aspect of family culture. However, as Richards (1997) points out, the lay notions of biological inheritance implied by such everyday discussion of family resemblances tend to sit in tension with newer ideas about inheritance derived from the genetics clinic. When it comes to genetic illnesses, ‘Family members try to make sense of the pattern of occurrence of the disorder they can observe in their family in terms of their previously held knowledge about inheritance’ (ibid. p. 267), with ‘visible phenotypic resemblances (physical, mental, and/or emotional) apparently also signify[ing] shared genotype or internal similarities including risk for disease’ (Chilibeck et al., 2011, p. 1771; see also Finkler et al., 2003).

Indeed, linking their child’s genetic condition to broader familial characteristics is so deep seated a desire that, as Dimond (2014) highlights, even in the case of a de novo genetic condition (which is, by definition, the result of a novel mutation and not inherited from either parent), parents seek to provide a familial explanation for the mutation, linking features of a child’s illness or behaviour to elements of a parent’s phenotype (see also McLaughlin & Clavering, 2011). In her work, exploring parents’ experience of testing for 22q11 deletion syndrome, Dimond notes that in the accounts of the parents in de novo cases, who therefore themselves did not test positive for the variant in question:

there remained a tendency to contextualise their child’s diagnosis within their own family history of health and illness… In constructing an explanation of the syndrome, parents seek familiarity. In this instance, the mother recognised that the syndrome was due to a ‘fluke of nature’ yet continued to contextualise this within her family history. Similarities were found between the son’s heart condition and the father’s angina.

(Dimond, 2014, pp. 155–156).

On the face of it, such accounts of disease causation and inheritance sit uncomfortably with scientific understandings of genetic disease. Indeed, as Chilibeck, Lock and Sehdev note, such lay approaches to genetics, incorporating as they do folk models of inheritance, run the risk of being ‘understood to represent a misunderstanding of the science’ (2011, p. 1770) or even, as Richards (1993) puts it, as ‘unscientific or irrational’ (p. 576).

Drawing on the ways in which genetics professionals themselves engage in similar explanatory habits of mind—expanding and speculating about parental phenotypes—we wish to suggest that seeking explanatory support from the appearance of pathology in parents is an almost unavoidable aspect of clinical decision-making around genetic illness. Indeed, in the context of new genomic techniques and related bioinformatics, it has become a real requirement. A crucial driver for this is the way in which different contributory criteria are taken into account when deciding whether a VUS can be classed as pathogenic or not. The decision over a variant’s VUS status is the result of ‘adding up’ different criteria of various strength (for a detailed discussion see Hedgecoe et al., 2023). The way in which such criteria are marshalled and combined means that information about parental phenotype and genotype can serve, in combination, as the ‘deciding vote’ in these decisions and thus as the key factor in deciding whether a VUS is inherited or has arisen de novo and, therefore, whether or not it can account for the pathology.

Turning to broader debates about the role of genomics in the clinic, at one level, the analysis presented in this article around the familialisation of genomics and the use of family history as an explanatory resource supports the position that emphasises the limited impact of genetic or genomic testing on clinical practice. While the results of such tests—be they clinical exome or whole genome sequences, or targeted sequence panels—are clearly important in these decisions, they do not take precedence over or ‘trump’ the phenotypic data. For information about parental genomes to be useful in the interpretation of their child’s results, we need to know their phenotype: Are they also affected? The answer to this question will sometimes be readily apparent, but on other occasions, it may be more difficult, requiring subtlety, and careful assessment to arrive at a determination and direct access to parental phenotype gives authority in professional debates. While the desire to resolve a VUS and provide a clear genetic explanation pushes the potential expansion of what might be considered a qualifying phenotype (e.g., ‘low end of normal’ intelligence as a sign of Rett Syndrome) a parent’s phenotypic reality can prove to be an unavoidable stumbling block. Although ‘Genomic platforms… [may]…bear the threat or promise (depending on one’s point of view) of de-centring clinical decision-making’ (Bourret et al., 2011, p. 817), it is clear that, in clinical genomics *as applied to the range of conditions treated in the meetings we observed*, this threat (or promise) has yet to solidify. In such cases, decision-making resembles the clinician-oriented, visually-centred approach set out in previous scholarship in this area (cf Latimer et al., 2006; Shaw, 2003). This key insight into current practice is, we believe, rooted in the differing perspectives of the various professional groups involved in this activity; clinical geneticists look for familial resemblances (shared *phenotypes*) and try to determine whether these are innocent or contribute to (comprise part of) the pathology. Molecular scientists/bioinformaticians, however, look for familial resemblances (shared *variants*) and try to determine whether these are innocent or contribute to (comprise part of) the pathology. While the overall aim is shared—to see whether a molecular variant can be identified as relevant to the phenotypic features—there is an asymmetry of ascribed professional competence: Clinical geneticists have the right to express an opinion about the molecular facts as well as the possibly dysmorphic features, while the laboratory scientists cannot claim the same right to comment on the physical features.

In other contexts, the weight accorded to judgements about the patients’ phenotypes will be more complex and more variable, especially when specialists from other disciplines may be present in addition to clinical and laboratory geneticists. Although we do not present the data here, a good example is provided by the MDT meetings to discuss families with inherited cardiac conditions. Here, the cardiologist will often have a much stronger ‘phenotypic voice’ in the interpretation of the clinical history and of any cardiac investigations performed than would the clinical geneticist; but at the same time, their ability to comment on the interpretation of molecular findings will be much less, unless they have gained specific molecular expertise (e.g., if they have worked in a research laboratory while gaining a Ph.D.). Both generic factors, such as the general level of molecular genetic competence within a specialty, and individual factors, such as that person’s skills and experiences, will mould the contribution they can make to the negotiations in these clinical-laboratory MDT negotiations.

While we could conclude our paper at this point, we feel that our data requires a further step, cautioning about generic claims regarding the relationship between clinical practice and the genomic laboratory. Our caution starts with the obvious response to above claims, emphasising that while claims centralising the role of genomics might not hold water in those areas where genomic technologies are most in use (i.e., where genetic inheritance plays a role in causation), there are other settings, such as the pharmacogenomic testing for oncology, as explored by Cambrosiso, Bourret and their colleagues, where such claims may indeed be accurate. Thus it may be that, contra the instinct in some STS writing to assume that new technologies lead to revolutions in practice (Hedgecoe & Martin, 2007), the focus in the genomic clinic should be on the conditions being tested rather than the technological innovations being introduced. For example, in discussing the role of clinical decision-making and molecular testing in cystic fibrosis, Joëlle Vailly draws an explicit distinction from dysmorphology, pointing out that the wider variety of symptoms and genetics in dysmorphology means one cannot always establish a link between them, in contrast to cystic fibrosis where, although the severity of the symptoms can vary, they are associated with a clearly identified and analysable gene making the approach simpler (even if ambiguities remain); as a result, the relative importance of the clinic as opposed to genetics decreases (Vailly, 2008). What is ‘normal’ in an oncology MDT (and thus the point of comparison) is clearly very different from a possible case of Rett Syndrome, where clinicians have to decide when a ‘kind of low average intelligence’ stops being part of the normal and becomes phenotypically suggestive.

In the same way as Timmermans and Haas (2008) critique sociology’s tendency to move from empirical data on specific disease populations to vague, generic discussion around, for example, ‘chronic illness’, we suggest that sociologists (and colleagues in related disciplines such as STS) need to be cautious about moving from a detailed exploration of the relationship between genetic testing and clinical practice in individual diseases to broad generalisations about ‘sequencing technology’ or ‘genomic testing’, or generic claims about the relationship between the clinic and the lab.

## Figures and Tables

**Table 1:** Summary of meetings observed.

Prenatal testing meetings: *n* = 87 + 2 ad hoc	Genomics MDT: 22
Medical genetics cases: 70	Cancer risk review: 25
Dysmorphology clinic meetings: 37	Cancer molecular meeting: 10
Developmental delay research meetings: 12	Inherited cardiac disease: 25

Abbreviation: MDT, multidisciplinary team.

## Data Availability

Ethnographic data, based on recordings, is not available since it renders participants identifiable and thus breaks ethics approval.

## Endnotes

1 The patient-facing element of our research was also extensive, involving observation and recording of 30 clinical consultations where the results of genetic/genomic tests were fed back to patients/family members. However, this current paper focuses on the internal professional discussions and thus does not draw on this dataset.

2 The growths associated with Cowden can often be found inside the mouth.

3 For an in-depth exploration of the Rett syndrome genetic clinic, see Featherstone & Atkinson, 2014.

4 The variable nature of X chromosome skewing means that different tissues in the same person will display differing levels of chromosomal activation.

5 Latimer (2013) traces the trope of parents ‘not coping’ to ‘clinical genetics’ roots in child and reproductive health care, and the practices of midwives and health visitors when making home visits during and after pregnancy.

6 A tightness of the muscles resulting from damage to the central nervous system.

7 That is, the nasal septum.

8 Talon cusps are additional bumps or layers on teeth.
